# Effects of grape seed extract supplementation on the growth performance, nutrients digestion and immunity of weaned lambs

**DOI:** 10.3389/fvets.2024.1402637

**Published:** 2024-09-13

**Authors:** Jian Ma, Tao Li, Lu Lin, Yuezhang Lu, Xi Chen, Sibing Li, Chunmei Du, Chen Wei, Fuquan Yin, Shangquan Gan

**Affiliations:** College of Coastal Agricultural Sciences, Guangdong Ocean University, Zhanjiang, China

**Keywords:** grape seed extract, lamb, growth performance, nutrient digestibility, immunity

## Abstract

Grape seed extract (GSE) has a variety of biological functions. At present, there has been limited information on the utilization of GSE as a feed additive in weaned lambs. The aim of this experiment was to study the potential influence of dietary supplementation with GSE on the growth performance, rumen fermentation characteristics, apparent digestibility, blood parameters and immunity in weaned lambs. In total, 30 male Hu sheep lambs with similar body weight (15.43 ± 0.49 kg) and age (48 ± 2 days) were randomly divided into two treatments: control (CON, fed basal ration) and GSE [fed basal ration and 0.6 g/d GSE (main compositions: proanthocyanidin 50%, catechin 24%, gallic acid 16% and epicatechin 6%) per lamb]. The feeding experiment lasted for 60 d. Results showed that GSE supplementation significantly increased (*p* = 0.008) the average daily gain of lambs. Compared with CON group, the ruminal propionate and butyrate concentrations were significantly increased (*p* < 0.05) in GSE group, whereas the ammonia nitrogen was decreased (*p* = 0.007). Also, the crude protein, neutral detergent fiber and ether extract digestibility of GSE group were higher (*p* < 0.05) than those of CON group. The serum contents of glucose, triglyceride, immunoglobulin G, glutathione peroxidase and total antioxidant capacity were significantly increased (*p* < 0.05) in GSE group when compared to those in CON group. However, an opposite trend of urea nitrogen, non-esterified fatty acid, interleukin-1β, itumor necrosis factor-α and malondialdehyde was observed between the two groups. Additionally, supplementation of GSE increased (*p* < 0.05) the *Lactobacillus* and decreased (*p* < 0.05) the *Escherichia coli* and *Salmonella* counts in the feces of lambs. In summary, GSE supplementation can improve growth performance, nutrient digestion and immunity of weaned lambs.

## 1 Introduction

In recent years, the rising demand of meat consumption has greatly stimulated the development of livestock industry, particularly in mutton sheep farming. Mutton plays an essential role in the global food and nutrition security because it is an important food source with high quality protein, fat, vitamins and mineral elements for the humans (1, 2). In some parts of the world, due to the limited land resources and urgent demand for meadow ecological conservation and restoration, the sheep production has gradually shifted from traditional grazing regime to modern large-scale intensive breeding (3). Nevertheless, because of space limitation and feedstuff changes, the intensive feeding regime causes some adverse effects, such as decreased resistance to oxidative stress. Moreover, the low growth performance as well as high mortality and morbidity of lambs seriously restrain the stable development of mutton sheep breeding industry (4, 5). Lamb stage is a vital period that can affect the future productivity of adult sheep. Early weaning can decrease the feeding cost and promote the digestive tract development of lambs to a certain degree, and meanwhile, early weaning decreases the length of reproductive cycle in sheep production (6). After weaning, the feeding method and feed type of lambs are changed. Due to the immature immune system of lambs, early weaning stress has an obviously adverse influence on the growth rate and health (6). Additionally, the gastrointestinal tracts of lambs are in the developmental phase and have low resistance to the changes of external environment (7). Because of this, weaned lambs are vulnerable to harmful bacteria which can lead to diarrhea and mortality (5). Therefore, relieving weaning stress and improving healthy growth of lambs have important significance in large-scale intensive feeding of mutton sheep production.

The utilization of plants extracts has attracted widely attention in the livestock and poultry industries (8–12). Plant extracts including polyphenol, saponin, flavone, sterol and essential oils have shown to improve the nutrient digestion, immunity and antioxidant ability, which have beneficial effects on productivity and health of animals (13–17). As a by-product from grape processing, grape seed extract (GSE) is extracted from grape seed and mainly includes proanthocyanidin, epicatechin gallate, gallic acid and epicatechin (18). Recently, the use of GSE is more and more extensive since it has multiple characteristics including anti-inflammatory, antibacterial, antioxidative and anti-diabetic abilities (19). With the prohibition of antibiotics and some drug feed additives, the grape seed products have been used in animal production. A previous study found that dietary supplementation with 1% grape seed and grape marc meal extract did not have obvious influence on the dry matter intake (DMI) of dairy cows but increased the milk yield (20). In finishing lambs, high-concentrate diet supplemented with grape seed proanthocyanidins at the level of 20 mg/kg body weight (BW) daily increased DMI and average daily gain (ADG) as well as improving meat quality (21). Because of being full of polyphenols, the grape seed products (supplemented level was 50 mg/kg BW) have been demonstrated to improve antioxidant and anti-inflammatory abilities of preweaning calves, which were beneficial for attenuating oxidative stress caused by heat stress (22). Moreover, the results of an *in vitro* study showed that GSE can relieve inflammatory reaction through reducing the expression levels of interleukin-1β (IL-1β), IL-6 and tumor necrosis factor-α (TNF-α) (23). A recent research in dairy calves reported that the addition of GSE in diet at the dosage of 50 mg/kg BW promoted the growth of dairy calves suffered from heat stress by improving energy metabolism, endocrine system and gut fermentation (24).

The healthy development of gastrointestinal tracts acts as a critical role in the feedstuff change of young ruminants from liquid to solid, which has important effect on nutrient digestion and growth performance of animals (7). As a unique digestive organ of ruminants for nutrient digestion and volatile fatty acid (VFA) absorption, the health and development of rumen are strongly associated with VFA production, especially butyrate and propionate (25). A previous research *in vitro* found that grape seed product had the ability to regulate VFA production and improve rumen fermentation (26). After weaning, the young ruminants undergo obvious stress and are vulnerable to harmful bacteria, resulting in reduced growth rate, antioxidant ability and immunity (27). As mentioned above, previous studies in finishing lambs, dairy cows and calves verified that dietary supplementation with grape seed products alleviated oxidative stress and inflammation, and enhanced antioxidant ability and immunity, which were helpful for improving growth performance (20–22, 24). However, at present, the information of the role of GSE as a feed additive in weaned lambs remains scarce. Based on previous studies, we hypothesized that dietary supplementation of GSE may improve the rumen fermentation, nutrient digestibility and immunity of weaned lambs. Therefore, the current experiment was conducted to study the effects of GSE on the growth performance, nutrient digestion, antioxidant ability and immunity of weaned lambs.

## 2 Materials and methods

### 2.1 Experimental design and animals management

In this study, 30 male Hu sheep lambs with similar age (48 ± 2 days) and BW (15.43 ± 0.49 kg) after weaning were selected. The lambs were labeled with ear mark and randomly assigned to one of two groups with 15 lambs each. One group was used as control with no GSE supplementation (CON), and the other group was GSE group which was supplemented with GSE (Beisong Plant Technology Co., Ltd., Xi’an, China; main components: proanthocyanidin 50%, catechin 24%, gallic acid 16% and epicatechin 6%). The additive amount of GSE was 0.6 g/d per lamb which was based on previous research in finishing lambs (21).

All experimental lambs were housed in 30 hutches with 1 lamb in each hutch. The lambs were provided a same basal diet twice daily at 09:00 and 17:00 respectively, permitting 5 to 10% orts, and had *ad libitum* access to drink clean water. The feeding trial lasted for 70 d, consisting of 10 d of adaptive phase, followed by 60 d of experimental period. At morning feeding, GSE was fully mixed with the experimental diet to feed lambs to ensure complete intake. The experimental diet was formulated according to the NRC (28) and the nutritional level of diet was in accordance with NRC standard. Feed compositions and nutritional levels of diet are described in Table 1.

**Table 1:** Feed compositions and nutritional levels of basal diet (DM basis).

Ingredients, %	Content	Nutrient levels	Content
Alfalfa hay	29.60	DM (%)	91.08
Corn straw	14.08	EE (%)	2.75
Whole corn silage	26.32	CP (%)	14.42
Corn	15.02	NDF (%)	32.06
Wheat bran	5.10	ADF (%)	20.44
Soybean meal	6.16	Ca (%)	0.78
Cottonseed meal	3.12	P (%)	0.41
Stone powder	0.22	ME^2^ (MJ/kg)	10.08
NaCl	0.13		
Premix^1^	0.25		

DM, dry matter; EE, ether extract; CP, crude protein; NDF, neutral detergent fiber; ADF, acid detergent fiber; ME, metabolizable energy.

1 The premix provided following per kilogram of diet: Fe 80 mg, Zn 40 mg, Mn 30 mg, Cu 8 mg, I 0.30 mg, Se 0.20 mg, Co 0.10 mg, VA 980 IU, VD 110 IU, VE 20 IU.

2 ME was a calculated value; the other nutrient levels were measured values.

### 2.2 Growth performance measurement

Before morning feeding on d 0 and 60, the electronic scale was used to determine the BW of lambs. The ADG was calculated from initial and final BW. Feed intake of each lamb was recorded based on the difference between daily feed offered and orts, and subsequently transformed into DMI. The feed efficiency of lambs was obtained through dividing DMI by ADG.

### 2.3 Samples collection of blood, rumen fluid and feces

Before morning feeding on d 0 and 60, the blood of all lambs was collected from the jugular vein by using vacuum tubes without any additive (Kangcai Medical Equipment Manufacturing Co., Ltd., Guangzhou, China). Next, blood samples were used to obtain serum by centrifugation at 3200 rpm for 12 min. Serum samples were stored in centrifugal tubes at −20°C for following analysis. In addition, rumen fluid samples of lambs (30 mL) were collected using a stomach tube (Huazhi Kaiwu Technology Co., Ltd., Chengdu, China) on d 60 at 4 h after morning feeding. After pH measurement of rumen fluid with a pH meter, the samples were filtered with nylon cloth, and then preserved in centrifuge tubes at −20°C for determination of rumen fermentation.

Fecal samples of each lamb were collected from d 54 to 59 by nylon sieve plates under the floor of individual hutch. During the collection of fecal samples, feed offered and refused were collected for each lamb. The fecal samples were thoroughly mixed for 6 consecutive days and weighed. Then, 10% of total feces was collected for following procedures. The fresh feed, orts and feces were mixed by each lamb, subsampled, and dried at 65°C to obtain a constant weight. Next, all samples were smashed through a 1-mm screen (Jinzhen Machinery manufacturing Co., Ltd., Xinxiang, China) for analysis of apparent digestibility. Moreover, a part of fecal samples of each lamb were preserved in sterile tubes at −80°C for measurement of microbial counts.

### 2.4 Measurement of rumen fermentation and serum parameters

The rumen fluid samples were thawed firstly, and then centrifuged at 15000 rpm for 10 min to obtain supernatant. The collected supernatant samples were used to analyze rumen fermentation characteristics including VFA (29), ammonia nitrogen (NH_3_-N) (30), and microbial protein (MCP) (31). Before measurement of serum parameters, the samples were thawed and fully mixed. The contents of biochemical indicators including alanine transaminase (ALT), aspartate transaminase (AST), alkaline phosphatase (ALP), triglyceride (TG), non-esterified fatty acid (NEFA), glucose (GLU), urea nitrogen (UN), total protein (TP), albumin (ALB), and globulin (GLB) in serum were analyzed by utilizing a biochemistry analyzer. The serum antioxidant parameters including glutathione peroxidase (GSH-Px), malondialdehyde (MDA), catalase (CAT), superoxide dismutase (SOD) and total antioxidant capacity (T-AOC) were measured with commercial kits (Nanjing Jiancheng Bioengineering Institute, Nanjing, China). Besides, the contents of serum immunoglobulins and cytokines including IL-1β, IL-6, IL-10, TNF-α, immunoglobulin M (IgM), IgA and IgG were determined by commercial kits (Nanjing Jiancheng Bioengineering Institute, Nanjing, China) following the procedures of specifications.

### 2.5 Measurement of nutrient digestibility and microbial count

The contents of DM (method 934.01), organic matter (OM, method 942.05), EE (method 954.02) and CP (method 984.13) in fresh feed, orts and feces were analyzed following the methods of AOAC (32). Furthermore, the NDF and ADF contents in these samples were measured using an Ankom fiber analyzer. With nutrients contents in feces and feed, the apparent digestibility (AD) was calculated by an equation as follows: AD (%) = [(nutrient intake − nutrient content in feces)/nutrient intake] × 100.

Plate method was used to count the *Lactobacillus*, *Bifidobacterium*, *Escherichia coli*, and *Salmonella* in the feces of lambs according to procedures of previous studies (33, 34). Briefly, in aseptic console, 10 g of fecal samples of each lamb were dissolved in 90 mL of sterile water and vibrated to blend well. Then, the mixed liquid was serially diluted to enumerate the microbial composition. The small protrusions that formed on the MRS plate with slightly white wet and neat edges were *Lactobacillus*. The double concentric ring colonies that formed a central bulge on the BBL plate were regarded as *Bifidobacterium*. Moreover, small colonies with purplish black and metallic luster forming on the eosin methylene blue plate were *Escherichia coli*. The colonies with black center forming on SS medium were deemed to be *Salmonella*.

### 2.6 Statistical analysis

Each lamb was regarded as experimental unit to analyze data. After checking the normality and homogeneity of variance tests, the data including growth performance, rumen fermentation, apparent digestibility, blood indexes and microbial count were analyzed through the independent samples t-test of SPSS software (version 22.0). Results are shown as means and standard error of mean. Significant difference was declared at *p* < 0.05, and a tendency was indicated at 0.05 < *p* < 0.01.

## 3 Results

### 3.1 Growth performance

Effects of GSE supplementation on the growth performance of weaned lambs are presented in Table 2. There was no significant difference (*p* > 0.05) of initial BW and DMI between the CON and GSE groups. However, dietary supplementation with GSE significantly increased (*p* < 0.05) final BW and ADG. In addition, compared with CON group, the feed efficiency was improved by 7.02% (*p* = 0.041) in GSE group.

**Table 2:** Effects of GSE supplementation on growth performance of lambs.

Items	Groups		SEM	*p*-value
	CON	GSE		
Initial BW (kg)	16.75	16.74	0.098	0.947
Final BW (kg)	27.26	28.81	0.373	0.035
ADG (g/d)	175.11	201.22	5.136	0.008
DMI (g/d)	896.29	950.67	20.762	0.195
Feed efficiency	5.13	4.77	0.089	0.041

GSE, grape seed tract; BW, body weight; ADG, average daily gain; DMI, dry matter intake; SEM, standard error of mean. CON, fed basal diet; GSE, fed basal diet and 0.6 g/d GSE per lamb.

### 3.2 Rumen fermentation

**Figure 1:**
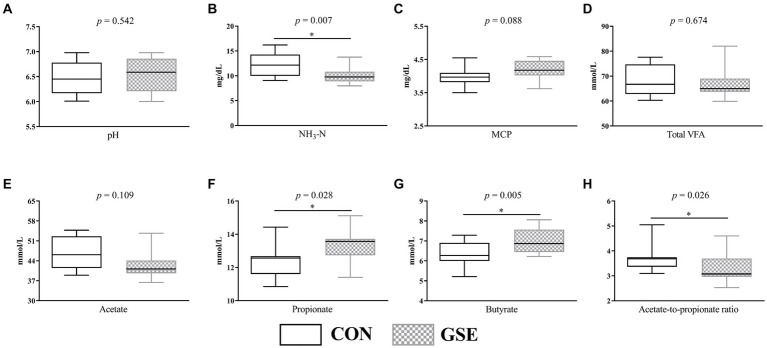
Effects of GSE supplementation on rumen fermentation characteristics of lambs. **(A)** pH; **(B)** NH_3_-N; **(C)** MCP; **(D)** total VFA; **(E)** acetate; **(F)** propionate; **(G)** butyrate; **(H)** acetate-to-propionate ratio. GSE, grape seed tract; NH_3_-N, ammonia nitrogen; MCP, microbial protein; VFA, volatile fatty acid. CON, fed basal diet; GSE, fed basal diet and 0.6 g/d GSE per lamb. The asterisk represents a significant difference (*p* < 0.05) between CON and GSE treatments.

As illustrated in Figure 1A, the average ruminal pH of CON and GSE group was 6.46 and 6.54 respectively, and did not show obvious difference (*p* > 0.05). Similarly, no obvious difference (*p* > 0.05) of ruminal total VFA (Figure 1D) and acetate (Figure 1E) concentrations was observed between the two groups. Nevertheless, GSE supplementation significantly reduced (*p* < 0.05) NH_3_-N content (Figure 1B). On the contrary, the ruminal propionate (Figure 1F) and butyrate (Figure 1G) concentrations of GSE group were higher (*p* < 0.05) than those of CON group. Compared with CON group, the ratio of acetate to propionate (Figure 1H) was decreased by 12.37% (*p* < 0.05) in GSE group. Moreover, the MCP concentration (Figure 1C) in GSE group tended to be higher (*p* = 0.088) than that in CON group.

### 3.3 Nutrient apparent digestibility

**Figure 2:**
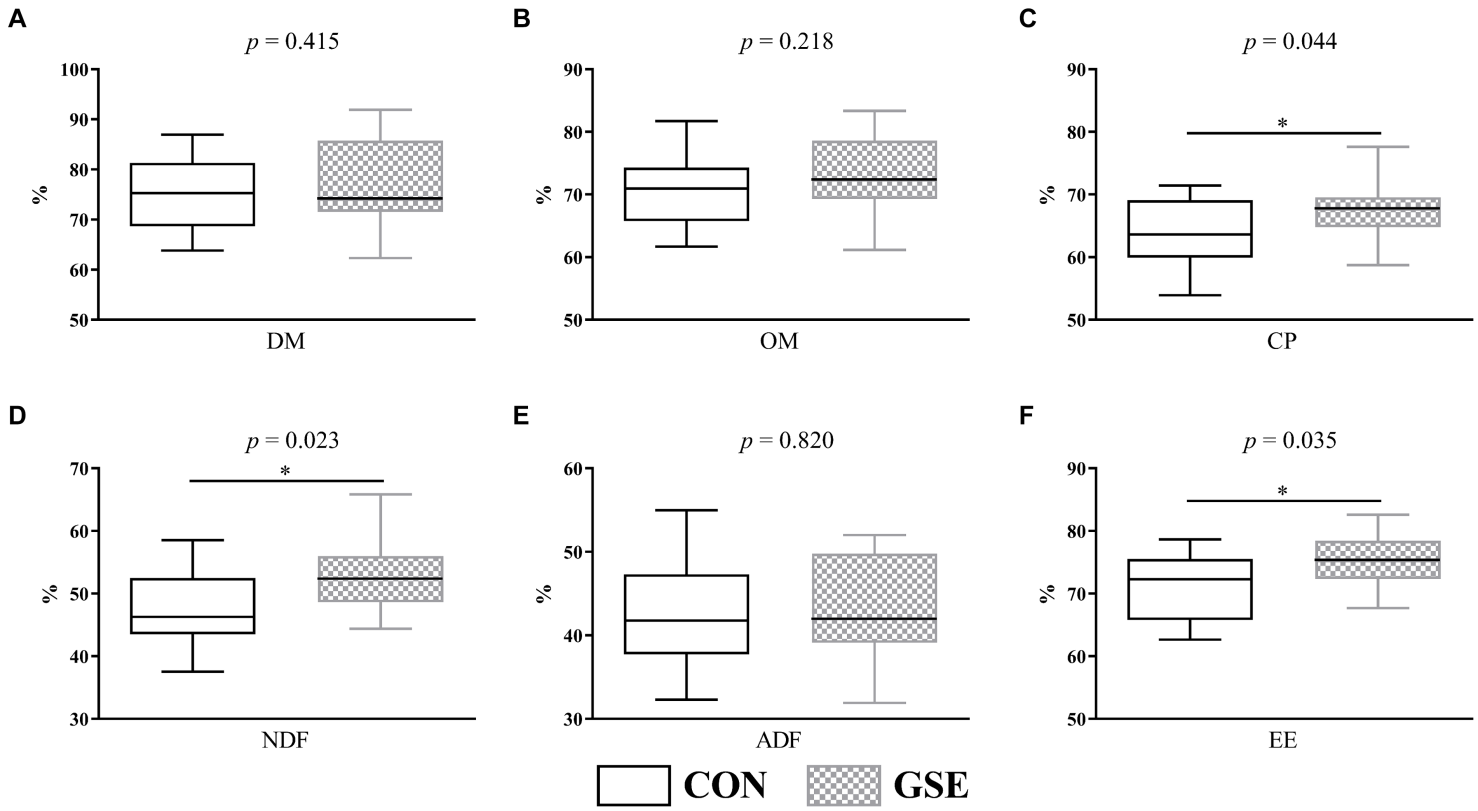
Effects of GSE supplementation on nutrient apparent digestibility of lambs. **(A)** DM; **(B)** OM; **(C)** CP; **(D)** NDF; **(E)** ADF; **(F)** EE. GSE, grape seed tract; DM, dry matter; OM organic matter; CP, crude protein; NDF, neutral detergent fiber; ADF, acid detergent fiber; EE, ether extract. CON, fed basal diet; GSE, fed basal diet and 0.6 g/d GSE per lamb. The asterisk represents a significant difference (*p* < 0.05) between CON and GSE treatments.

Obviously, the DM (Figure 2A), OM (Figure 2B) and ADF (Figure 2E) apparent digestibility were similar (*p* > 0.05) between the CON and GSE groups. However, lambs fed GSE had significantly increased (*p* < 0.05) CP (Figure 2C), NDF (Figure 2D) and EE (Figure 2F) digestibility.

### 3.4 Biochemical indicator of serum

On d 0, the concentrations of all biochemical indexes in serum of lambs were not different (*p* > 0.05) in CON and GSE groups (Table 3). Likewise, the serum contents of ALT, AST, ALP, TP, ALB and GLB were similar (*p* > 0.05) between the two groups on d 60. However, the TG and GLU contents of GSE group were higher (*p* < 0.05) than those of CON group, whereas an opposite trend was found in NEFA and UN contents between the two groups.

**Table 3:** Effects of GSE supplementation on biochemical indicators in serum of lambs.

Items	Groups		SEM	*p*-value
	CON	GSE		
Day 0				
ALT (U/L)	17.45	17.93	0.317	0.458
AST (U/L)	83.64	87.44	2.124	0.380
ALP (U/L)	254.41	262.17	7.575	0.617
TG (mmol/L)	0.382	0.369	0.006	0.292
NEFA (mmol/L)	0.169	0.162	0.010	0.726
GLU (mmol/L)	4.79	4.88	0.072	0.561
UN (mmol/L)	5.34	5.23	0.087	0.537
TP (g/L)	49.45	48.90	0.975	0.786
ALB (g/L)	27.26	25.97	0.893	0.478
GLB (g/L)	22.19	22.94	0.707	0.603
Day 60				
ALT (U/L)	16.88	17.46	0.382	0.459
AST (U/L)	88.59	87.46	1.787	0.758
ALP (U/L)	252.94	258.57	7.971	0.730
TG (mmol/L)	0.411	0.441	0.007	0.022
NEFA (mmol/L)	0.173	0.135	0.009	0.021
GLU (mmol/L)	4.89	5.15	0.052	0.009
UN (mmol/L)	4.99	4.72	0.057	0.014
TP (g/L)	52.49	51.92	0.972	0.776
ALB (g/L)	27.07	28.45	0.878	0.441
GLB (g/L)	25.42	23.47	0.796	0.227

GSE, grape seed tract; ALT, alanine transaminase; AST, aspartate transaminase; ALP, alkaline phosphatase; TG, triglyceride; NEFA, non-esterified fatty acid; GLU, glucose; UN, urea nitrogen; TP, total protein; ALB, albumin; GLB, globulin; SEM, standard error of mean. CON, fed basal diet; GSE, fed basal diet and 0.6 g/d GSE per lamb.

### 3.5 Serum immunoglobulin

No significant difference (*p* > 0.05) of IgM, IgA and IgG contents was found between the CON and GSE treatments on d 0 (Table 4). A similar tendency of IgM was observed between the two groups on d 60. Compared with CON group, the IgA and IgG concentrations in serum of lambs were increased by 12.12% (*p* = 0.068) and 10.00% (*p* = 0.026) in GSE group, respectively.

**Table 4:** Effects of GSE supplementation on immunoglobulin contents in serum of lambs.

Items	Groups		SEM	*p*-value
	CON	GSE		
Day 0				
IgM (mg/mL)	3.95	4.27	0.131	0.226
IgA (mg/mL)	21.19	22.22	0.632	0.425
IgG (μg/mL)	50.28	48.56	0.840	0.313
Day 60				
IgM (mg/mL)	4.32	4.67	0.175	0.322
IgA (mg/mL)	23.26	26.08	0.774	0.068
IgG (μg/mL)	51.22	56.34	1.172	0.026

GSE, grape seed tract; IgM, immunoglobulin M; IgA, immunoglobulin A; IgG, immunoglobulin G; SEM, standard error of mean; CON, fed basal diet; GSE, fed basal diet and 0.6 g/d GSE per lamb.

### 3.6 Serum cytokine

Table 5 shows the difference of cytokine concentrations between the CON and GSE groups. On d 0, there was no obvious difference (*p* > 0.05) in serum IL-1β, IL-6, IL-10 and TNF-α contents between the two groups. On d 60, dietary supplementation of GSE significantly reduced (*p* < 0.05) serum IL-1β and TNF-α concentrations in lambs. Besides, the IL-6 content in GSE treatment was slightly lower (*p* = 0.072) than that in CON group, while an opposite tendency of IL-10 was found between the two groups.

**Table 5:** Effects of GSE supplementation on cytokine contents in serum of lambs.

Items	Groups		SEM	*p*-value
	CON	GSE		
Day 0				
IL-1β (ng/L)	104.66	100.99	2.859	0.530
IL-6 (ng/L)	139.85	142.39	3.291	0.707
IL-10 (ng/L)	88.19	91.17	1.269	0.521
TNF-α (ng/L)	525.20	511.27	8.092	0.399
Day 60				
IL-1β (ng/L)	90.22	82.71	1.431	0.006
IL-6 (ng/L)	159.72	148.87	3.022	0.072
IL-10 (ng/L)	71.08	77.10	1.649	0.068
TNF-α (ng/L)	502.51	477.21	5.564	0.020

GSE, grape seed tract; IL-1β, interleukin-1β; IL-6, interleukin-6; IL-10, interleukin-10; TNF-α, tumor necrosis factor-α; SEM, standard error of mean; CON, fed basal diet; GSE, fed basal diet and 0.6 g/d GSE per lamb.

### 3.7 Antioxidant index of serum

The serum antioxidant parameters did not show a significant difference (*p* > 0.05) between the CON and GSE treatments on d 0 (Table 6). After GSE supplementation, the serum GSH-Px and T-AOC activities of lambs were significantly enhanced (*p* < 0.05), while the MDA concentration showed an opposite trend between the two groups. Moreover, a slight improvement (*p* = 0.054) of SOD concentration was found in GSE group when compared to CON group.

**Table 6:** Effects of GSE supplementation on antioxidant indexes in serum of lambs.

Items	Groups		SEM	*p*-value
	CON	GSE		
Day 0				
GSH-Px (U/mL)	126.64	122.48	3.354	0.544
MDA (mmol/mL)	4.97	5.11	0.153	0.638
CAT (U/mL)	53.71	56.03	1.197	0.341
SOD (U/mL)	65.12	68.13	1.684	0.382
T-AOC (U/mL)	10.51	10.32	0.197	0.648
Day 60				
GSH-Px (U/mL)	130.88	138.95	2.017	0.043
MDA (mmol/mL)	4.23	3.43	0.180	0.023
CAT (U/mL)	50.28	47.81	0.811	0.130
SOD (U/mL)	69.32	73.19	1.012	0.054
T-AOC (U/mL)	14.93	16.06	0.251	0.019

GSE, grape seed tract; GSH-Px, glutathione peroxidase; MDA, malondialdehyde; CAT, catalase; SOD, superoxide dismutase; T-AOC, total antioxidant capacity; SEM, standard error of mean; CON, fed basal diet; GSE, fed basal diet and 0.6 g/d GSE per lamb.

### 3.8 Microbial count of feces

**Figure 3:**
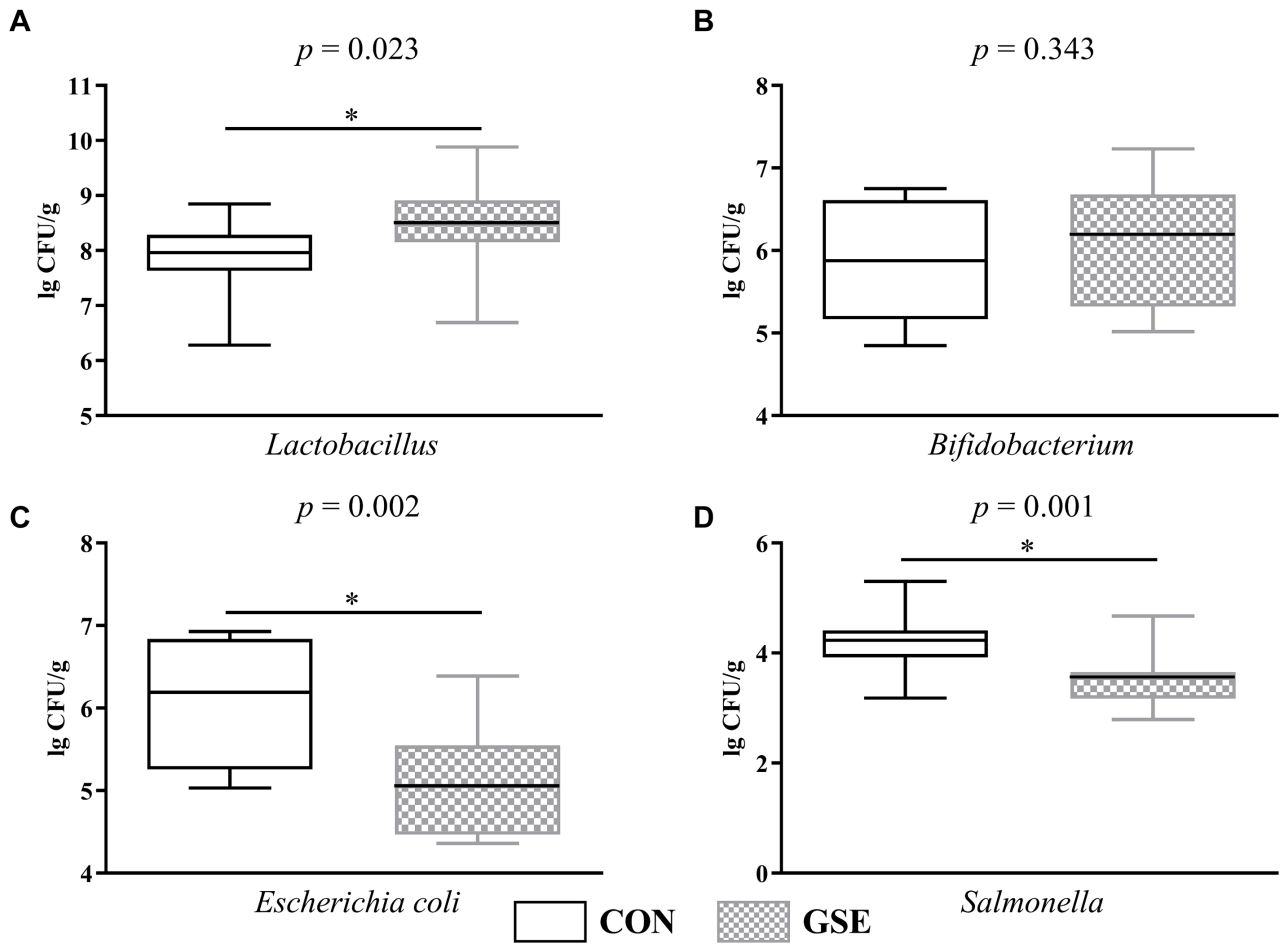
Effects of GSE supplementation on microbial count in feces of lambs. **(A)** *Lactobacillus*; **(B)** *Bifidobacterium*; **(C)** *Escherichia coli*; **(D)** *Salmonella*. GSE, grape seed tract. CON, fed basal diet; GSE, fed basal diet and 0.6 g/d GSE per lamb. The asterisk represents a significant difference (*p* < 0.05) between CON and GSE treatments.

Notably, dietary GSE supplementation significantly increased (*p* < 0.05) the *Lactobacillus* count in the feces of lambs (Figure 3A). The number of *Bifidobacterium* did not show significant difference (*p* > 0.05) between the CON and GSE groups (Figure 3B). Compared with CON group, dietary supplementation of GSE observably decreased (*p* < 0.05) the *Escherichia coli* (Figure 3C) and *Salmonella* (Figure 3D) counts in the feces of lambs.

## 4 Discussion

Lamb nutrition has attracted increasingly attention in the current sheep industry. The healthy rearing of lambs is of great significance for future growth rate and productivity of fattening sheep (35, 36). Because the gastrointestinal tracts and immune system are still in developmental stage, the lambs are extremely vulnerable to harmful microbe. After weaning, the lambs experience certain challenges, such as changes of feedstuff, feeding regime and physiological metabolism, leading to reduced growth rate and immunity as well as increased incidence rate (5, 37). Alleviating the weanling stress has important impacts on promoting rapid growth of lambs (36). GSE, as a green feed additive, has a variety of physiological functions, which include antioxidative, antibacterial and anti-inflammation effects (19). In fattening sheep, a research found that high-concentrate ration supplemented with grape seed proanthocyanidin increased the feed intake and weight gain (21). In the current experiment, the BW and ADG of lambs were obviously increased after dietary addition of GSE, but the feed intake did not display obvious change. The differences in research results may be that the developmental stage (post-weaning and fattening respectively) of experimental animals was distinct. Research result obtained from our experiment also showed that GSE supplementation significantly improved the feed conversion of lambs. Previously, an experiment in rabbits found that the feed conversion was ameliorated after GSE supplementation under condition of heat stress (38), which was consistent with our finding. The proanthocyanidin of grape seed can oxidize proteins on the surface of pathogenic bacteria, which can destroy the structure and function of protein molecules, resulting in bacterial death (39). The positive effect of proanthocyanidin mentioned earlier is conducive to inhibiting the growth of pathogenic bacteria, thus reducing morbidity and attenuating adverse influence on growth of lambs induced by weanling stress.

The normal fluctuation range of ruminal pH is from 6 to 7, and it is a vital index to visually judge the healthy condition of rumen (6). A recent research reported that low roughage ration supplemented with grape by-product reduced the ruminal pH (40). Unfortunately, we did not find obvious difference of ruminal pH between the CON and GSE groups. However, the pH values of two treatments ranged from 6.15 to 6.98, which partly suggested that GSE had no adverse impact on ruminal health. On the other hand, weaned lambs fed GSE reduced the ruminal NH_3_-N content, indicating that GSE could improve the utilization of NH_3_-N to a certain degree. Consistent with our study, a previous research found that polyphenols had the ability to decrease rumen NH_3_-N and increase MCP concentrations (41). The ruminal microorganisms can synthesize MCP using ammonia and other nutrients. Interestingly, the MCP content in the rumen of lambs was slightly increased after GSE supplementation, which corresponded to NH_3_-N result. In the rumen, supplementation of plant polyphenols is helpful for weakening protein degradation by ruminal microorganisms and enhancing absorbable dietary protein for the small intestine (42). The positive role of GSE in improving ruminal MCP may be attributed to potential advantage of polyphenols mentioned earlier.

The VFA is the main energy source to maintain the metabolism and productivity of ruminants, providing 70 to 80% of the energy requirements (43). In our experiment, the propionate and butyrate contents in the rumen of lambs were higher in GSE group than those in CON group. As an important energy source, the GLU required for metabolism in ruminants mainly comes from gluconeogenesis in the liver. Propionate is a primary precursor for liver gluconeogenesis (44). Hence, the increased propionate in the rumen might promote growth of lambs. Butyrate is an important energy substance for ruminal epithelium and can up-regulate gene expressions related to cell proliferation, which plays an essential role in stimulating ruminal development (25). In addition, an early research has confirmed that butyrate can accelerate mucosal tissue development through decreasing apoptosis (45). The increased butyrate concentration in the rumen after GSE supplementation might be beneficial for promoting ruminal development of lambs. We also found that dietary supplementation of GSE significantly decreased ruminal acetate-to-propionate, suggesting that a propionate type fermentation was occurred of lambs. Rumen propionate fermentation can improve fiber degradation and feed utilization (43), which provide more energy for lambs to promote growth. Previously, a research found that grape seed by-product increased the relative abundance of butyrate-producing bacteria using ruminal simulation technique (46), which contributed to higher ruminal content of butyrate. However, the influence of GSE on the ruminal microbiome of lambs still needs further exploration.

Due to the inadequate secretion of digestive enzyme, the nutrient apparent digestibility of newly weaned lambs is relatively lower (47). In the present study, the addition of GSE significantly increased CP, NDF and EE digestibility, which indicated that the lambs in GSE group can obtain more nutrients to promote growth. The ruminal microbes degrade diets to produce MCP and small peptides, and these substances can be easily absorbed by small intestine to enhance CP digestibility (48). We speculated that the improvement of CP digestibility by GSE supplementation might attributed to increased MCP content. Also, the NDF digestibility of GSE lambs was higher than CON lambs, which was in accordance with previous research by Juráček et al. (49), who found that wethers fed grape residue increased NDF digestibility. In a previous study of preweaning dairy calves, dietary supplementation with gallic acid, a component of GSE, has been verified to increase relative abundances of microorganisms associated with fiber degradation and VFA production in the rumen, which mainly included *Ruminococcaceae*, *Bacteroides* and *Christensenellaceae* (50). The higher NDF digestibility of GSE treatment was probably a connection with the regulation of gastrointestinal microbiome. Moreover, the increased EE digestibility of GSE lambs might have beneficial effects on the weight gain and fat deposition. Lastly, digestive enzyme, existed in the gut, has important function in improving nutrients digestibility. Therefore, future research should pay more attention to study the influence of GSE on the activity of digestive enzyme in weaned lambs.

Serum biochemical parameters are strongly linked with healthy condition of animals, and these biochemical parameters can reflect metabolic status of body (13, 49). The changes of ALT, AST and ALP concentrations in serum are vital indicators related to liver function, and GLU, TG and NEFA contents reflect lipid and energy metabolism. Furthermore, to some extent, the protein metabolism can be assessed by changes of serum TP and UN contents (24, 49). A previous study in lactating dairy ewes found that dietary supplementation of grape seed had no significant effects on the blood ALT and AST concentrations (51). In our experiment, no obvious difference of serum contents of ALT, AST and ALP was observed, indicating that dietary addition of GSE did not have adverse influence on the liver function of lambs. Nevertheless, results obtained from our experiment showed that the serum GLU and TG concentrations were significantly increased by GSE treatment, whereas GSE supplementation reduced serum NEFA concentration of lambs. The possible reason for improvement in energy metabolism of lambs is that GSE increases ruminal propionate content and accelerates gluconeogenesis process, thus resulting in increased serum GLU content. But the potential molecular mechanism requires further exploration. After undergoing stress such as heat, cold and weaning of animals, the TG in the adipose tissue will be decomposed into NEFA to provide energy for various metabolism through blood circulation (52). The negative effects on energy metabolism of lambs caused by weaning was improved by GSE treatment, which were conducive to improving growth of lambs. On the other hand, we found that GSE treatment significantly reduced serum UN content, indicating that the nitrogen conversion was higher in GSE lambs. The variation tendency in nitrogen conversion was basically consistent with CP digestibility, which suggested that the improved nitrogen conversion of GSE lambs could be attributed to the increased CP digestibility. A previous study in dairy cows reported that high concentrations of serum UN, milk urea nitrogen and ruminal NH_3_-N were negatively correlated with nitrogen efficiency (53). In this experiment, considering the low concentrations of ruminal NH_3_-N and serum UN in GSE lambs, an improved efficiency of nitrogen conversion should be expected. A great deal of nitrogen excreted by ruminants reduce the efficiency of feed utilization, and meanwhile, it cause environmental pollution (47). Dietary supplementation of GSE increased ruminal MCP content, which might be beneficial for improving nitrogen absorption in the small intestine of lambs. In the future, more data including nitrogen intake as well as fecal and urinary nitrogen excretion should be collected to further investigate the potential positive effects of GSE on the nitrogen conversion.

Serum immunoglobulins are a class of protein molecules with extensive immune functions, which can combine specifically to antigens, activate complement and bind to Fc receptors on the cell surface to obtain specific immunity (54). The serum concentrations of immunoglobulins can be used to reflect the immunity of animals. In our study, GSE supplementation increased serum IgG and IgA contents, indicating that GSE enhanced immunity of weaned lambs. The polyphenols can improve function of immune system and resistance of body to disease by promoting white blood cell activity, increasing antibody production and improving cellular immune response (55), which might explain the improvement of GSE on the immune function. Weaning stress of lambs often induces dysfunction of gastrointestinal epithelium, and then results in the release of pro-inflammatory factors, which finally causes inflammatory response (56). Our research showed that GSE supplementation significantly decreased the serum IL-1β and TNF-α of lambs, while increased IL-10 concentration. The IL-1β and TNF-α are main pro-inflammatory cytokines, and the increased concentrations are associated with inflammatory response (23). As an anti-inflammatory cytokine, IL-10 can down-regulate the expression of pro-inflammatory cytokines such as TNF-α, IL-6, and IL-1β by activating macrophages and inhibiting the combination between cytokines and receptors (57). The results of current study indicated that GSE relieved the inflammatory reaction of lambs, which contributed to healthy growth. A previous study in calves suffered from heat stress reported that GSE decreased plasma TNF-α content and alleviated inflammatory reaction (22), which was in accordance with our result. Another research reported that proanthocyanidin, a main ingredient of GSE, reduced the expression of pro-inflammatory factors through inhibiting NF-κB signaling pathway (58). In the future, more studies including *in vivo* and *in vitro* should be carried out to investigate the potential effects of NF-κB signaling pathway in attenuating inflammation by GSE.

Weaning is a source of stress for lambs and can cause oxidative stress (5). The MDA, an oxidative stress index, damage the cells integrity, and can be used to reflect the degree of injury for cell and tissues (59). The GSH-Px, SOD and T-AOC are important indexes to assess antioxidant activity of body, which can scavenge free radical and prevent macromolecule damage (59). In the current study, the GSH-Px, SOD and T-AOC activities in serum of lambs were enhanced by GSE treatment, while the MDA activity was reduced. Results from our study indicated that GSE supplementation increased the antioxidant ability of lambs. Previous experiments in fattening lambs, dairy calves and rabbits have demonstrated that grape seed products can increase the activity of antioxidant enzyme under stress condition (21, 22, 38), which were in line with current study. Plant polyphenol has been verified to play the role of antioxidation by Nr2/Keap1 signaling pathway (60), which may be responsible for the improvement in antioxidant ability of GSE. Taken together of serum indexes, GSE supplementation can effectively improve energy metabolism and antioxidant ability, and finally enhance immunity of weaned lambs.

Gut microorganism is an effective barrier against intestinal pathogens, which has important role in protecting intestinal tissues and maintaining the normal function of immune system (5). Weaning stress is accompanied by changes in the intestinal environment, which makes lambs vulnerable to the invasion of pathogens, resulting in diarrhea and other diseases (5). The *Escherichia coli* and *Salmonella* are the main pathogens that induce diarrhea and threaten the health of lambs (61). In this study, GSE supplementation significantly decreased the *Escherichia coli* and *Salmonella* counts, indicating that GSE can protect the gut of lambs against disease. A recent study report that GSE had obvious bacteriostasis for *Escherichia coli* (62), which was consistent with our result. The reason may be that the proanthocyanidins and other components contained in grape seed can effectively inhibit the growth of intestinal pathogenic bacteria. However, the specific mechanism still needs to be studied. The *Lactobacillus* can produce lactic acid in the intestine to maintain an acidic environment, and then prevent the colonization of other pathogens, thus protecting the intestinal health (63). In dairy calves, the increased *Lactobacillus* count has been verified to positively associate with high weight gain and feed efficiency (33). Our microbial results showed that GSE supplementation had the ability to protect gut health of lambs. This positive effect was also found in improved serum immune parameters of our research. Future experiments to investigate the functions of microbial community by metagenomics and host function by transcriptomics are required to obtain more results on the role of microbiome in the gut of lambs and their response to GSE.

## 5 Conclusion

The addition of GSE can improve the ADG and feed efficiency as well as CP, NDF and EE digestibility of weaned lambs. It also can improve rumen fermentation of weaned lambs, which are reflected by increased propionate and butyrate contents and decreased NH_3_-N concentration in the rumen. In addition, supplementation of GSE can enhance the antioxidant ability and immunity of weaned lambs by increasing the serum GSH-Px, T-AOC and IgG contents and fecal *Lactobacillus* count, and decreasing the serum MDA, IL-1β and TNF-α concentrations and fecal *Escherichia coli* and *Salmonella* counts. Therefore, GSE can be used as a feed additive of weaned lambs. These data provided theoretical support for the application of GSE in lamb husbandry.

## Data Availability

The raw data supporting the conclusions of this article will be made available by the authors, without undue reservation.

## References

[1] Vahmani P Ponnampalam EN Kraft J Mapiye C Bermingham EN Watkins PJ. Bioactivity and health effects of ruminant meat lipids. Invited review. Meat Sci. (2020) 165:108114. doi: 10.1016/j.meatsci.2020.108114, PMID: 32272342

[2] Ding W Lu Y Xu B Chen P Li A Jian F. Meat of sheep: insights into mutton evaluation, nutritive value, influential factors, and interventions. Agriculture. (2024) 14:1060. doi: 10.3390/agriculture14071060

[3] Huang Y Liu L Zhao M Zhang X Chen J Zhang Z. Feeding regimens affecting carcass and quality attributes of sheep and goat meat — a comprehensive review. Anim Biosci. (2023) 36:1314–26. doi: 10.5713/ab.23.0051, PMID: 37402458 PMC10472155

[4] Zhang H Zheng Y Zha X Liu X Ma Y Loor JJ. Dietary N-carbamylglutamate and L-arginine supplementation improves redox status and suppresses apoptosis in the colon of intrauterine growth-retarded suckling lambs. Anim Nutr. (2022) 11:359–68. doi: 10.1016/j.aninu.2022.08.009, PMID: 36329684 PMC9618968

[5] Guo H Cui J Li Q Liang X Li J Yang B. A multi-omic assessment of the mechanisms of intestinal microbes used to treat diarrhea in early-weaned lambs. mSystems. (2024) 9:e0095323. doi: 10.1128/msystems.00953-23, PMID: 38193712 PMC10878098

[6] Abdelsattar MM Zhao W Saleem AM Kholif AE Vargas-Bello-Pérez E Zhang N. Physical, metabolic, and microbial rumen development in goat kids: a review on the challenges and strategies of early weaning. Animals. (2023) 13:2420. doi: 10.3390/ani13152420, PMID: 37570229 PMC10417166

[7] Li Y Guo YL Zhang CX Cai XF Liu P Li CL. Effects of physical forms of starter feed on growth, nutrient digestibility, gastrointestinal enzyme activity, and morphology of pre-and post-weaning lambs. Animal. (2021) 15:100044. doi: 10.1016/j.animal.2020.100044, PMID: 33516036

[8] Ghazwani M Hakami AR Sani SS Sultana S Sultana T Bashir W. Antibacterial activity of aqueous and methanolic extract of *Mentha piperita* against pervasive bacteria isolated from urial the *Ovis vignei*. Pak Vet J. (2023) 43:103–8. doi: 10.29261/pakvetj/2023.018

[9] Tchoupou-Tchoupou EC Ndofor-Foleng HM Nwenya JM Okenyi NJ Ikeh Nnana E Ngwu NR. Effects of hexane extract of garlic on hematological, biochemical and histological parameters in F1 crossbred chicks non-infected and infected with *Salmonella typhimurium*. Int J Vet Sci. (2022) 11:435–42. doi: 10.47278/journal.ijvs/2022.135

[10] Xu D Wang X Shi W Bao Y. *Lonicera flos* and *Curcuma longa* L. extracts improve growth performance, antioxidant capacity and immune response in broiler chickens. Front Vet Sci. (2024) 11:1388632. doi: 10.3389/fvets.2024.1388632, PMID: 38681856 PMC11045969

[11] Nawaz M Zhou J Khalid I Shamim A Hussain A Ahmed Z. Antiparasitic activity of plants extract against gastrointestinal nematodes and *Rhipicephalus microplus*. Int J Vet Sci. (2022) 11:474–8. doi: 10.47278/journal.ijvs/2022.147

[12] Velázquez-Antunez J Olivares-Perez J Olmedo-Juárez A Rojas-Hernandez S Villa-Mancera A Romero-Rosales T. Biological activity of the secondary compounds of *Guazuma ulmifolia* leaves to inhibit the eggs *Haemonchus contortus* hatching. Pak Vet J. (2023) 43:55–60. doi: 10.29261/pakvetj/2022.075

[13] Rehman A Hussain K Zaman MA Faurk MAZ Abbas A Mero WMS. Effect of coneflower, neem, and thyme extracts on growth performance, blood chemistry, immunity and intestinal microbial population of broilers. Kafkas Univ Vet Fak. (2023) 29:407–13. doi: 10.9775/kvfd.2023.29625

[14] Aljohani ASM. Botanical compounds: a promising approach to control *Mycobacterium* species of veterinary and zoonotic importance. Pak Vet J. (2023) 43:633–42. doi: 10.29261/pakvetj/2023.107

[15] Coelho MG da Silva AP de Toledo AF Cezar AM Tomaluski CR Barboza RDF. Essential oil blend supplementation in the milk replacer of dairy calves: performance and health. PLoS One. (2023) 18:e0291038. doi: 10.1371/journal.pone.0291038, PMID: 37788273 PMC10547158

[16] Caliskan GU Emin N. Protective efficacy of fresh and aged macerated garlic oils in safflower oil against intra-abdominal adhesions in rats. Pak Vet J. (2023) 43:290–6. doi: 10.29261/pakvetj/2023.030

[17] Ali W Khatyan U Sun J Alasmari A Alshahrani MY Qazi IH. Mitigating effect of pomegranate peel extract against the furan induced testicular injury by apoptosis, steroidogenic enzymes and oxidative stress. Chemosphere. (2024) 358:142086. doi: 10.1016/j.chemosphere.2024.142086, PMID: 38670510

[18] Silva JTP Borges MH de Souza CAC Fávaro-Trindade CS Sobral PJA de Oliveira AL. Grape pomace rich-phenolics and anthocyanins extract: production by pressurized liquid extraction in intermittent process and encapsulation by spray-drying. Food Secur. (2024) 13:279. doi: 10.3390/foods13020279, PMID: 38254580 PMC10814744

[19] Dimitrina RK Yavor I Zlatina RC Tzonka G. Antimicrobial potential, antioxidant activity, and phenolic content of grape seed extracts from four grape varieties. Microorganisms. (2023) 11:395. doi: 10.3390/microorganisms11020395, PMID: 36838361 PMC9963647

[20] Gressner DK Koch C Romberg FJ Winkler A Dusel G Herzog E. The effect of grape seed and grape marc meal extract on milk performance and the expression of genes of endoplasmic reticulum stress and inflammation in the liver of dairy cows in early lactation. J Dairy Sci. (2015) 98:8856–68. doi: 10.3168/jds.2015-9478, PMID: 26409958

[21] Mu C Yang W Wang P Zhao J Hao X Zhang J. Effects of high-concentrate diet supplemented with grape seed proanthocyanidins on growth performance, liver function, meat quality, and antioxidant activity in finishing lambs. Anim Feed Sci Technol. (2020) 266:114518. doi: 10.1016/j.anifeedsci.2020.114518

[22] Urkmez E Biricik H. Grape seed extract supplementation in heat-stressed preweaning dairy calves: I. Effects on antioxidant status, inflammatory response, hematological and physiological parameters. *Animl feed*. Sci Technol. (2022) 292:115421. doi: 10.1016/j.anifeedsci.2022.115421

[23] Nallathambi R Poulev A Zuk JB Raskin I. Proanthocyanidin-rich grape seed extract reduces inflammation and oxidative stress and restores tight junction barrier function in caco-2 colon cells. Nutrients. (2020) 12:1623. doi: 10.3390/nu12061623, PMID: 32492806 PMC7352846

[24] Urkmez E Biricik H. Grape seed extract supplementation in heat-stressed preweaning dairy calves: II. Effects on growth performance, blood metabolites, hormonal responses, and fecal fermentation parameters. Anim Feed Sci Technol. (2022) 292:115422. doi: 10.1016/j.anifeedsci.2022.115422

[25] Li Z Wang X Wang W An R Wang Y Ren Q. Benefits of tributyrin on growth performance, gastrointestinal tract development, ruminal bacteria and volatile fatty acid formation of weaned small-tailed Han lambs. Anim Nutr. (2023) 15:187–96. doi: 10.1016/j.aninu.2023.08.006, PMID: 38023378 PMC10679854

[26] Thanh LP Kha PTT Loor JJ Hang TTT. Grape seed tannin extract and polyunsaturated fatty acids affect *in vitro* ruminal fermentation and methane production. J Anim Sci. (2022) 100:skac039. doi: 10.1093/jas/skac039, PMID: 35137104 PMC8919818

[27] Hulbert LE Moisa SJ. Stress, immunity, and the management of calves. J Dairy Sci. (2016) 99:3199–216. doi: 10.3168/jds.2015-10198 26805993

[28] National Research Council. Nutrient requirements of small ruminants. Washington, DC: The National Academies Press (2007).

[29] Erwin ES Marco GJ Emery EM. Volatile fatty acid analyses of blood and rumen fluid by gas chromatography. J Dairy Sci. (1961) 44:1768–71. doi: 10.3168/jds.S0022-0302(61)89956-6

[30] Broderick GA Kang JH. Automated simultaneous determination of ammonia and total amino acids in ruminal fluid and *in vitro* media. J Dairy Sci. (1980) 63:64–75. doi: 10.3168/jds.S0022-0302(80)82888-8, PMID: 7372898

[31] Makkar HPS Sharma OP Dawra RK Negi SS. Simple determination of microbial protein in rumen liquor. J Dairy Sci. (1982) 65:2170–3. doi: 10.3168/jds.S0022-0302(82)82477-6, PMID: 7153399

[32] AOAC. Official Methods of Analysis. Washington, DC: Association of Official Analytical Chemists (2019).

[33] Alimirzaei M Alijoo YA Dehghan-Banadaky M Eslamizad M. The effects of feeding high or low milk levels in early life on growth performance, fecal microbial count and metabolic and inflammatory status of Holstein female calves. Animal. (2020) 14:303–11. doi: 10.1017/S1751731119001691 31368430

[34] Sanders ER. Aseptic laboratory techniques: plating methods. J Vis Exp. (2012) 63:3064. doi: 10.3791/3064-v PMC4846335 22617405

[35] Flinn T Kleemann DO Swinbourne AM Kelly JM Weaver AC Walker SK. Neonatal lamb mortality: major risk factors and the potential ameliorative role of melatonin. J Animal Sci Biotechnol. (2020) 11:107. doi: 10.1186/s40104-020-00510-w, PMID: 33292527 PMC7643391

[36] Zhao F He W Wu T Elmhadi M Jiang N Zhang A. Supplementation of coated sodium butyrate relieved weaning stress and reshaped microbial flora in weaned lambs. Front Vet Sci. (2024) 11:1423920. doi: 10.3389/fvets.2024.1423920, PMID: 39104550 PMC11299240

[37] Mao HL Ji WW Yun Y Zhang YF Li ZF Wang C. Influence of probiotic supplementation on the growth performance, plasma variables, and ruminal bacterial community of growth-retarded lamb. Front Microbiol. (2023) 14:1216534. doi: 10.3389/fmicb.2023.1216534, PMID: 37577421 PMC10413120

[38] Hassan FA Mahrose KM Basyony MM. Effects of grape seed extract as a natural antioxidant on growth performance, carcass characteristics and antioxidant status of rabbits during heat stress. Arch Anim Nutr. (2016) 70:141–54. doi: 10.1080/1745039X.2016.1139609, PMID: 26829476

[39] Andersone A Janceva S Lauberte L Ramata-Stunda A Nikolajeva V Zaharova N. Anti-inflammatory, anti-bacterial, and anti-fungal activity of oligomeric proanthocyanidins and extracts obtained from lignocellulosic agricultural wast. Molecules. (2023) 28:863. doi: 10.3390/molecules28020863, PMID: 36677921 PMC9861313

[40] Suescun-Ospina ST Vera N Astudillo R Yunda C Williams P Allende R. Effects of País grape marc inclusion in high and low forage diets: ruminal fermentation, methane production and volatile fatty acids. Ital J Anim Sci. (2022) 21:924–33. doi: 10.1080/1828051X.2022.2076620

[41] Puchalska J Szumacher-Strabel M Patra AK Ślusarczyk S Gao M Petrič D. The effect of different concentrations of total polyphenols from *Paulownia* hybrid leaves on ruminal fermentation, methane production and microorganisms. Animals. (2021) 11:2843. doi: 10.3390/ani11102843, PMID: 34679864 PMC8532658

[42] Theodorou MK Kingston-Smith AH Winters AL Lee MRF Minchin FR Morris P. Polyphenols and their influence on gut function and health in ruminants: a review. Environ Chem Lett. (2006) 4:121–6. doi: 10.1007/s10311-006-0061-2

[43] Gäbel G Sehested J. SCFA transport in the forestomach of ruminants. Comp Biochem Physiol. (1997) 118:367–74. doi: 10.1016/S0300-9629(96)00321-0, PMID: 9366072

[44] Aschenbacah JR Kristensen NB Donkin SS Hammon HM Penner GB. Gluconeogenesis in dairy cows: the secret of making sweet milk from sour dough. Int Union Biochem Mol Biolo Life. (2010) 62:869–77. doi: 10.1002/iub.400, PMID: 21171012

[45] Mentschel J Leiser R Mülling C Pfarrer C Claus R. Butyric acid stimulates rumen mucosa development in the calf mainly by a reduction of apoptosis. Arch Anim Nutr. (2001) 55:85–102. doi: 10.1080/17450390109386185 12068484

[46] Khiaosa-ard R Metzler-Zebeli BU Ahmed S Muro-Reyes S Deckardt K Chizzola R. Fortification of dried distillers grains plus solubles with grape seed meal in the diet modulates methane mitigation and rumen microbiota in Rusitec. J Dairy Sci. (2015) 98:2611–26. doi: 10.3168/jds.2014-8751, PMID: 25648805

[47] Zhang W Sun S Zhang Y Zhang Y Wang J Liu Z. Benzoic acid supplementation improves the growth performance, nutrient digestibility and nitrogen metabolism of weaned lambs. Front Vet Sci. (2024) 11:1351394. doi: 10.3389/fvets.2024.1351394, PMID: 38406631 PMC10884225

[48] Ma J Zhu Y Wang Z Yu X Hu R Wang X. Glutamine supplementation affected the gut bacterial community and fermentation leading to improved nutrient digestibility in growth-retarded yaks. FEMS Microbiol Ecol. (2021) 97:fiab084. doi: 10.1093/femsec/fiab084, PMID: 34132351

[49] Juráček M Vašeková P Massányi P Kováčik A Bíro D Šimko M. The effect of dried grape pomace feeding on nutrients digestibility and serum biochemical profile of wethers. Agriculture. (2021) 11:1194. doi: 10.3390/agriculture11121194

[50] Xu HJ Zhang QY Wang LH Zhang CR Li Y Zhang YG. Growth performance, digestibility, blood metabolites, ruminal fermentation, and bacterial communities in response to the inclusion of gallic acid in the starter feed of preweaning dairy calves. J Dairy Sci. (2022) 105:3078–89. doi: 10.3168/jds.2021-20838, PMID: 35086717

[51] Nudda A Correddu F Marzano A Battacone G Nicolussi P Bonelli P. Effects of diets containing grape seed, linseed, or both on milk production traits, liver and kidney activities, and immunity of lactating dairy ewes. J Dairy Sci. (2015) 98:1157–66. doi: 10.3168/jds.2014-8659, PMID: 25497793

[52] Moiseeva K Anipchenko P Vasil’eva S Karpenko LY Vasil’ev R Pilaeva N. Dynamics of cholesterol and triglycerides in the serum of cows with liver lipidosis. J Anim Sci. (2019) 97:208. doi: 10.1093/jas/skz258.42730351413

[53] Nousiainen J Shingfield KJ Huhtanen P. Evaluation of milk urea nitrogen as a diagnostic of protein feeding. J Dairy Sci. (2004) 87:386–98. doi: 10.3168/jds.S0022-0302(04)73178-1 14762082

[54] Yin X Ji S Duan C Tian P Ju S Yan H. The succession of fecal bacterial community and its correlation with the changes of serum immune indicators in lambs from birth to 4 months. J Integr Agr. (2023) 22:537–50. doi: 10.1016/j.jia.2022.08.055

[55] Hong M Cheng L Liu Y Wu Z Zhang P Zhang X. A natural plant source-tea polyphenols, a potential drug for improving immunity and combating virus. Nutrients. (2022) 14:550. doi: 10.3390/nu14030550, PMID: 35276917 PMC8839699

[56] Izuddin WI Loh TC Foo HL Samsudin AA Humam AM Postbiotic L. *Plantarum* RG14 improves ruminal epithelium growth, immune status and upregulates the intestinal barrier function in post-weaning lambs. Sci Rep. (2019) 9:9938. doi: 10.1038/s41598-019-46076-0, PMID: 31289291 PMC6616331

[57] Al-Ashy B Chakroun I El-Sabban ME Homaidan FR. The role of NF-κB in mediating the anti-inflammatory effects of IL-10 in intestinal epithelial cells. Cytokine. (2006) 36:1–8. doi: 10.1016/j.cyto.2006.10.003, PMID: 17161612

[58] Terra X Pallarès V Ardèvol A Bladé C Fernandez-Larrea J Pujadas G. Modulatory effect of grape-seed procyanidins on local and systemic inflammation in diet-induced obesity rats. J Nutr Biochem. (2011) 22:380–7. doi: 10.1016/j.jnutbio.2010.03.006, PMID: 20655715

[59] Du X Cheng X Ji K Degen AA Liang Y Wu X. Effects of supplementary thyme on immunity responses, antioxidant indices, rumen enzymes concentrations and rumen bacteria composition in Hu sheep. Anim Feed Sci Technol. (2023) 306:115828. doi: 10.1016/j.anifeedsci.2023.115828

[60] Huang T Che Q Chen X Chen D Yu B He J. Apple polyphenols improve intestinal antioxidant capacity and barrier function by activating the Nrf2/Keap1 signaling pathway in a pig model. J Agr Food Chem. (2022) 70:7576–85. doi: 10.1021/acs.jafc.2c02495, PMID: 35679090

[61] Tuo X Wang S Cui D Wang F Wang H Liu Y. Antibiotic resistance profiles and virulence markers of *Escherichia coli* strains isolated from diarrheal lambs in Gansu and Qinghai, China. Pak Vet J. (2020) 40:123–6. doi: 10.29261/pakvetj/2019.102

[62] Yaqubi AK Astuti SD Zaidan AH Syahrom A Nurdin DZI. Antibacterial effect of red laser-activated silver nanoparticles synthesized with grape seed extract against *Staphylococcus aureus* and *Escherichia coli*. Lasers Med Sci. (2024) 39:47. doi: 10.1007/s10103-024-03991-7, PMID: 38277009

[63] Kahrstrom CT Pariente N Weiss U. Intestinal microbiota in health and disease. Nature. (2016) 535:47. doi: 10.1038/535047a 27383978

